# Influence of the Artificial Aging Heat Treatment Regime on the Cavitation Erosion Behavior of the AM50 Alloy

**DOI:** 10.3390/ma19132826

**Published:** 2026-07-02

**Authors:** Ilare Bordeasu, Dorin Bordeasu, Filip-Sebastian Tatu, Daniel-Catalin Stroita, Cristian Ghera

**Affiliations:** 1 Department of Mechanical Machines, Equipment and Transports, Politehnica University of Timisoara, Bulevardul Mihai Viteazul nr. 1, 300222 Timisoara, Romania; ilare.bordeasu@upt.ro; 2 Department of Automation and Applied Informatics, Politehnica University of Timisoara, Vasile Parvan, No. 2, 300223 Timisoara, Romania; 3 Clinica de Ortopedie si Trautomatologie, Spital Clinic Militar de Urgenta “Dr. Victor Popescu” Timisoara, Str. Gheorghe Lazăr nr. 7, 300080 Timisoara, Romania; filiptatu@gmail.com

**Keywords:** magnesium alloy, structural state, mass loss, erosion rate, resistance to cavitation, artificial aging heat treatment, microstructure, hardness

## Abstract

The use of bulk heat treatments to improve the resistance of the material structures to cavitation erosion remains an effective approach due to the beneficial modifications induced in the microstructure and physical-mechanical properties. Depending on the intensity of cavitation loading, various heat treatment regimes can be applied. Among these, artificial aging treatments are particularly suitable for non-ferrous alloys, especially aluminum, zinc, and magnesium-based alloys. The current study investigates the effect of artificial aging heat treatment performed at 250 °C with holding times of 12 and 24 h on the biodegradable magnesium-based AM50 alloy. Cavitation tests were carried out using the method with a stationary specimen on a standard vibratory device according to ASTM G32-2016 requirements. The analysis of cavitation-eroded surfaces through macro- and microstructural images, together with the interpretation of characteristic erosion curves and specific parameters (cumulative mass loss, erosion speed and cavitation resistance), revealed both similarities and significant differences governed primarily by surface hardness and microstructural features. Comparison with the initial (semi-finished) state and with previous studies on artificial aging treatments performed at 200 °C for 12 and 24 h confirms the similarly beneficial effect of the 250 °C aging regime on the cavitation erosion resistance of the AM50 alloy.

## 1. Introduction

Non-ferrous metal alloys with low specific mass, such as those based on aluminum and magnesium, are known, in particular, for their applications in the automotive, aeronautical, military, sports and medical fields. Their extension to the manufacture of parts that work in specific conditions of cavitation hydrodynamics has required the development of research on their behavior under these erosive stresses. If, for aluminum-based alloys used in the manufacture of parts that work in cavitational currents such as boat propellers, pump rotors, pistons and valves of thermal engines, the literature [1,2,3,4,5] offers many research results regarding their behavior and resistance to cavitation erosion, for magnesium-based alloys, research in this direction is in its early stages. Most studies related to magnesium-based alloys are due to their biodegradability and use in reconstructive surgery and orthopedic devices [5,6,7,8,9,10,11,12,13,14,15,16,17,18,19,20].

The expansion of the use of these alloys in cardiac surgery devices (stents, valves), due to the blood network, of the hydraulic network type, requires the need to study them also from the point of view of behavior under stresses generated by cavitation [21,22,23,24,25,26]. These studies aim to identify the mechanism of destruction of the surface structure, at overpressures of the order of tens, even hundreds of GPa, created by the impact with microjets and shock waves produced by the implosion of cavitation bubbles [27,28,29,30,31,32,33,34,35]. Significant is the research carried out by Krelka et al. [21] who shows that the mechanism of cavitation degradation of pure magnesium and its alloys is ductile fracture. Also, Krella [23], through the research carried out on the AlMg_2_ alloy, highlighted the negative effect of ductile intermetallic compounds, by increasing the speed and depth of the caverns produced by cavitation erosion.

Aquire et al. [36] conducted a study on the formation of caverns by cavitation in the structure of the AZ61 alloy, finding that the process begins at the grain boundaries, being preceded by a strong plastic deformation and, over time, develops perpendicular to the direction of stress.

Jascionowski et al. [24] in their research, carried out on the MgAl_2_Si alloy, in three stands with different intensities of cavitation destruction (vibrating apparatus, hydrodynamic tunnel and pressurized jet), shows the dependence of the degree of destruction on the type of stand, practically expressing the dependence of erosion on the intensity of the hydrodynamic regime of cavitation. The studies conducted by YE L et al. [22] on the AZ31B alloy, using a vibrating apparatus with the indirect method (stationary sample), show the effect of the liquid medium (water and kerosene) on the increase in the Vickers hardness of the surface, under the impact of microjets and shock waves, generated by the mechanism of vibratory cavitation. The authors’ conclusions are that cavitation in water increased the Vickers hardness by (23.77–48.19)% compared to that obtained by cavitation in kerosene. It is known that the main purpose of research in the field of cavitation is to increase the service life of parts. The most used technologies for this purpose are volumetric heat treatments, due to the possibilities of varying the parameters of the regime: temperature, holding time, cooling medium. An example in this regard is the research conducted on various structural states of pure zinc [8,25,37,38,39,40,41,42,43] and its alloys ZnMg [6,9,37,44], ZnCuMg [8,10,45], also targeting applications in cardiovascular surgery. The results of these research studies highlight the effect of the parameters of the artificial aging heat treatment regime through the changes brought to the microstructure (the basic solid solution, the secondary phases and the brittle intermetallic compounds) and the values of the mechanical properties of the alloy. While bulk heat treatments are the most commonly applied methods for tailoring the microstructure and mechanical performance of materials intended for cavitation-exposed components, considerable research efforts have also focused on the application of surface coating technologies as an effective means of improving surface resistance to cavitation erosion [46,47,48].

Through the results reported herein, this study contributes to the growing database of research on biodegradable alloys and offers novel insights into the influence of artificial aging heat treatment regimes conducted at 250 °C for 12 and 24 h on the cavitation erosion behavior and erosion resistance of the AM50 magnesium alloy.

## 2. The Researched Material: Heat Treatment Regimes

The material studied is the AM50 magnesium-based alloy cast under pressure, in two structural states resulting from the volumetric heat treatment regimes of artificial aging at 250 °C, with holding times of 12 and 24 h. For each regime, cooling was in air. After casting and prior to the artificial aging treatments, the bars from which the cavitation test specimens were machined were subjected to a stress-relief heat treatment at 150 °C for 2 h, followed by slow furnace cooling. The chemical composition of the alloy in the initial condition (as-cast semi-finished product) was: 4.70% Al, 0.32% Mn, 0.13% Zn, 0.002% Fe, 0.001% Ni, 0.004% Cu, 0.03% Si, 0.0014% Be, 94.77% Mg, and 0.005% other elements. This composition, together with the mechanical properties reported in Refs. [49,50], was determined in the specialized laboratories of the Politehnica University of Timișoara, Romania.

**Figure 1:**
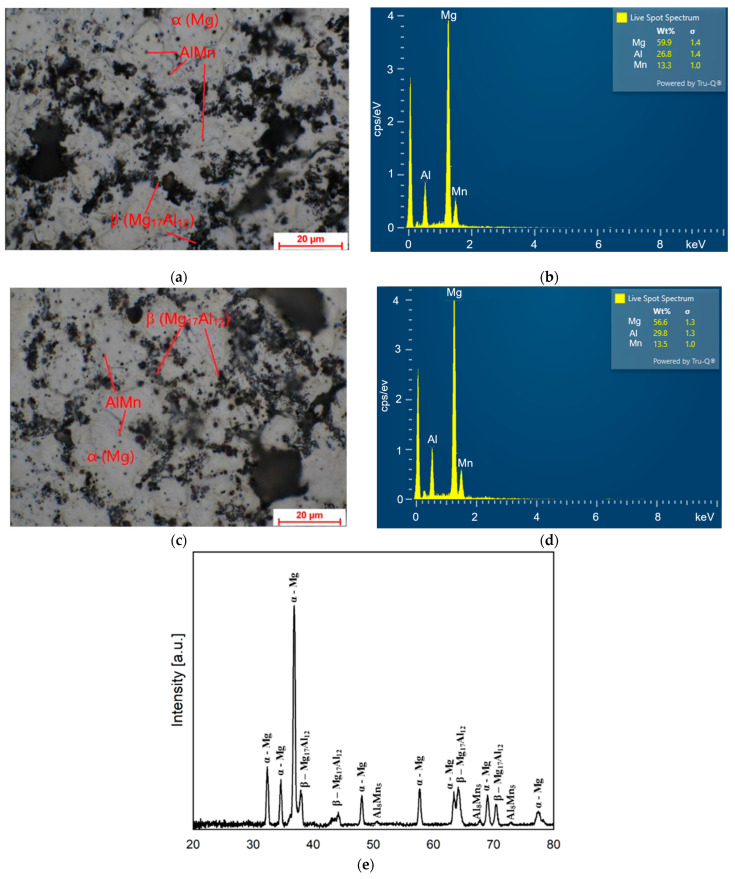
Microstructural image (6% nital attack) and energy-dispersive X-ray (EDX) spectrum of the surfaces structures before cavitation exposure and surface structure diffractograms before attack. (**a**) Microstructural image of 250/12 h state. (**b**) The EDAX image of the 250/12 h state of β phase. (**c**) Microstructural image of 250/24 h state. (**d**) The EDAX image of the 200/24 h state of β phase and (**e**) Surface diffractogram.

For simplification, in the presentation of experimental results, discussions and analyses, the notion of state (structural!) and the symbols that suggest the applied heat treatment regime are used, as follows:

- 250/12 h—structural state resulting from artificial aging heat treatment at 250 °C with a holding time of 12 h;
- 250/24 h—structural state resulting from artificial aging heat treatment at 250 °C with a holding time of 24 h;

In Figure 1 are shown the images of the microstructures, energy-dispersive X-ray (EDX) spectrum and X-ray diffraction (XRD) pattern of the investigated surfaces before cavitation exposure, which is identical for both structural states. The microstructural images are obtained with an OLYMPUS metallographic microscope equipped with image processing software (Olympus Corporation, Hamburg, Germany) at the Physical Metallurgy laboratory of the National University of Science and Technology Politehnica Bucharest. For the diffractogram, the PANalytical X’Pert diffractometer (Malvern Panalytical, Bucharest, Romania) with Cu Kα radiation (λ = 1.5406 Å) was used. The angular range 2θ, in which the measurements are made, is between 20° and 80°, at a step of 0.03° and an integration time of 1 s/step. The tube voltage and current were 45 kV and 30 mA.

The EDX spectra presented in Figure 1b,d allowed the local chemical composition of the β phase in the AM50 alloy to be determined. For both the 250/12 h and 250/24 h aging conditions, the β phase contained approximately 61% Mg, 10 w% Mn, and 29% Al. These results, together with the XRD analysis, confirm the presence of the α(Mg), β(Mg_17_Al_12_), and Al_8_Mn_5_ phases in the investigated alloy. As the main objective of the present work is to assess the cavitation erosion behavior and resistance imparted by the applied heat treatment regimes, and because the volume fraction of secondary phases is lower than 3%, a detailed quantitative EDS characterization of the constituent phases was considered beyond the scope of this study.

The microscopic images (SEM), EDX spectra and the diffractogram in Figure 1 show differences between the concentrations and sizes of the intermetallic compound Al_8_Mn_5_ and the basic solid solutions α(Mg) and secondary β(Mg_17_Al_12_). From the diffractogram it is observed that the predominant phase is the basic solid solution α-Mg. There are also low-intensity reflections of the intermetallic phase β-Mg_17_Al_12_, formed at the boundaries between the grains and peaks of the brittle intermetallic compound Al_8_Mn_5_.

The fact that the diffractogram is identical for both states results in the conclusion that the significant differences, which also determine differences in their behaviors and resistances to the cyclic stresses of vibratory cavitation, are determined by: structural stability, the distribution of the secondary phase β(Mg_17_Al_12_), the intermetallic compound Al_8_Mn_5_, the degree of fineness and hardness value. Microscopic analyses show that the 250/24 h structural state presents a more refined microstructure and a higher level of crystalline defects, compared to the 250/12 h state.

From the studies carried out on aluminum-based alloys [1,2,3,4,23,24,50,51,52] and zinc-based alloys [4,39,53] in various structural states, it follows that an important role in the degree of destruction of the structure and its resistance to overpressures of impact with cavitational microjets is played by the intermetallic phases. From our microscopic measurements regarding the dimensions of these phases, slight variations were noted between the two structural states: from 5 μm to 35 μm in the 250/12 h state and from 5 μm to 30 μm for the 250/24 h state. The size of these particles was assessed using optical metallography and SEM analysis. Based on the studies carried out on aluminum-based alloys and zinc-based alloys, we appreciate that these dimensional variations and dispersions are effects of the heat treatment regimes.

Studies by Hobbs [54], Garcia [27] and Hammitt [28] on various ferrous and aluminum alloys show that surface hardness is the mechanical property with the greatest influence on the resistance of the alloy structure to overpressures, of the order of tens/hundreds of GPa [31,32,33,34,35], developed by microjets and shock waves upon impact with the surface exposed to cavitation. Therefore, because surface hardness influences the behavior of the surface structure to cavitation attack, and the morphology of destruction by caverns and rupture, eight measurements were made on one sample from each condition with the Zwick 3212 Hardness Tester (Zwick Roell, Germany). The algebraic mean values are: 61 HV_5_ (±2.5 HV_5_) for the 250/12 h condition and 64 HV_5_ (±2 HV_5_) for the 250/24 h condition.

## 3. Research Equipment and Experimental Results

**Figure 2:**
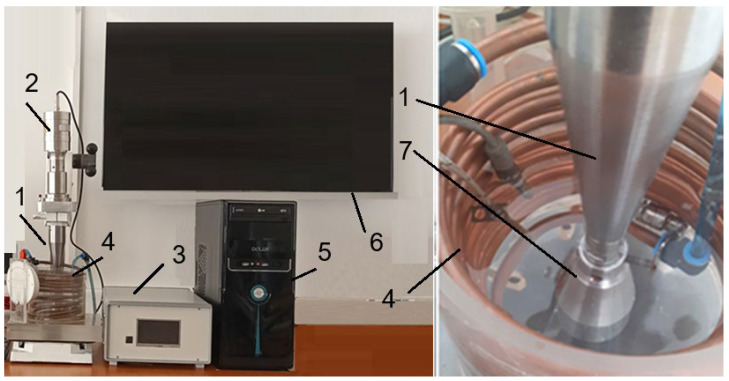
Cavitation testing apparatus: 1—sonotrode; 2—piezoceramic transducer 20 kHz; 3—electronic ultrasound generator; 4—the vessel with liquid and the cooling coil; 5—computer for controlling functional parameters; 6—monitor for tracking operating parameters; 7—fixing device for sample.

The cavitation tests were carried out in the Cavitation Erosion Research Laboratory of the Politehnica University of Timisoara, on the vibrating device with piezoceramic crystals, Figure 2. The functional parameters, which dictate the intensity of the cavitation regime, are: power of the electronic ultrasound generator = 500 W, the vibration amplitude = 50 μm, vibration frequency = 20 ± 0.03 kHz, diameter of the surface exposed to cavitation = 15.8 mm, and liquid medium = distilled water at 22 ± 1 °C). The vibrating device is equipped with a computer with software for controlling the functional parameters of the device (vibration amplitude and frequency, power of the electronic ultrasound generator) and a refrigerator for maintaining the water temperature within the allowed limits.

The testing procedure was performed using a stationary sample (indirect method) [12,52,55] which is in accordance with the requirements of the international standard ASTM G32-2016 [56]. According to the custom in the field, used by most researchers, for certainty, three samples were tested from each condition. The preparation of the samples for testing, the total duration of the attack, the intermediate periods at which determinations of the lost mass are made, the examinations and photographic records of the surface exposed to cavitation, and the recording and processing of experimental data are according to the custom of the laboratory [55].

For the analysis of the morphology of the surface degradation, by the erosion of vibrating cavitation, after the completion of the test, the scanning electron microscope TESCAN VEGA 3 LMU Bruker EDX Quantax and the computer tomography TESCAN Unitom HR (Tescan, Brno, Czech Republic) were used. For investigations of the degradation evolution, as an extension, at the end of each intermediate cavitation period, the Canon Power Shot A 480 camera was used.

### 3.1. The Morphology of Structural Degradation

**Figure 3:**
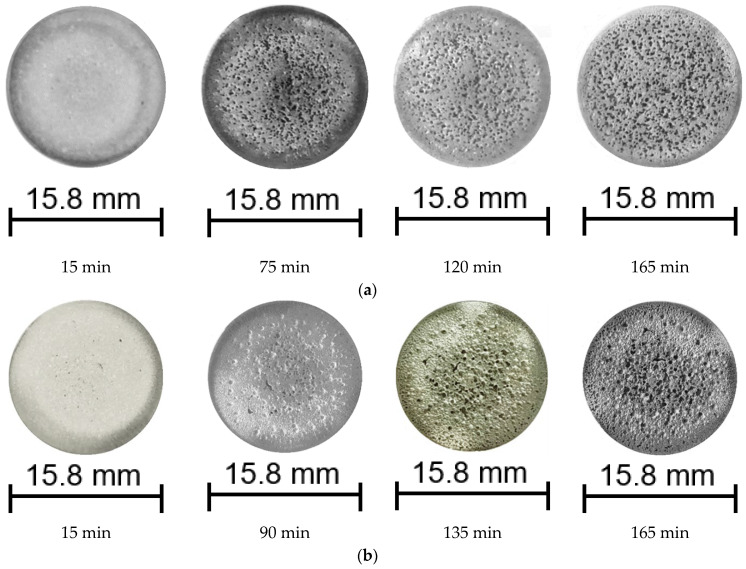
The evolution of cavitation erosion in the attacked surface (photographic images taken with the Canon Power Shot A 480 camera) of the: (**a**) 250/12 h state; (**b**) 250/24 h state.

**Figure 4:**
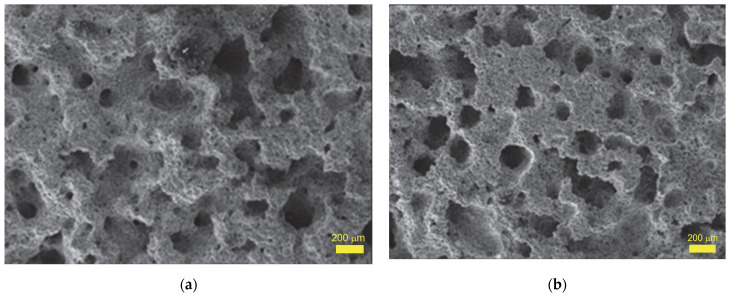
The SEM images of the caverns in the eroded surface of the: (**a**) 250/12 h (×100) state; (**b**) 250/24 h (×100) state.

**Figure 5:**
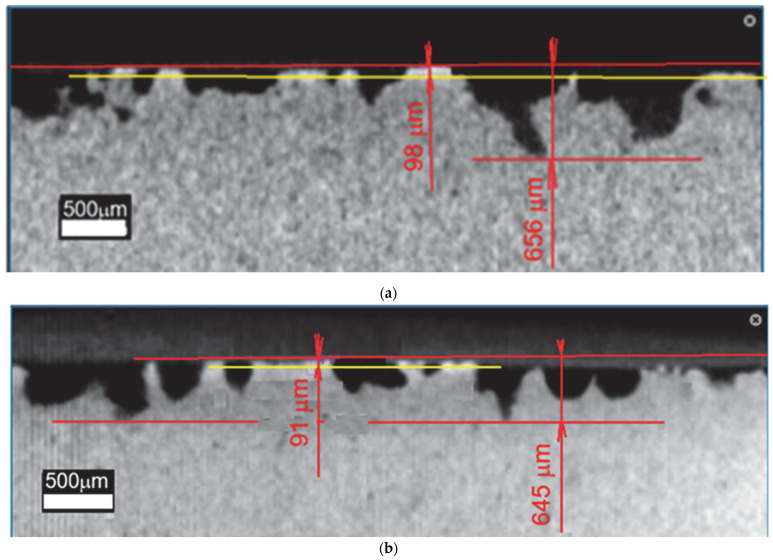
The CT images of the cavernous sinus depth of the: (**a**) 250/12 h state; (**b**) 250/24 h state.

For morphological analysis, macro (photographic) images are presented in Figure 3 from four significant periods, and SEM images (Figure 4) and CT (Figure 5) after the completion of the cavitation attack (165 min). For microscopic investigation with the acquisition of images in Figure 4, the TESCAN VEGA 3 LMU Bruker EDX Quantax electron microscope was used. To illustrate the depth of the caverns in Figure 5, in an arbitrary axial plane (perpendicular to the eroded area), the computer tomography procedure with the Panthera 1.4.4 software and the TESCAN Unitom HR computer tomography were used. The images in Figure 3 show slightly different evolutions of the cavern formation in the eroded area. These differences are the effect of the heat treatment regime, on the grain sizes of the basic solution α, of the secondary phase β and on the dispersion and sizes of the brittle AlMn compounds, which, according to studies carried out on aluminum alloys [1,2,3,4,23,24,50,51,52], zinc alloys [6,7,8,42,45,49] and on AlMg_2_ [23], are the first to yield to cyclic stresses of the microjets, representing primers for cracks and caverns. It should be noted that visual differences, by the area of the eroded surface, by the shape and number of microcavities, which express a higher resistance of the structure of the 250/24 h state, are noticeable up to 90 min of cavitation stress. After this time, until the end of the test, both structures have identical behavior in terms of the degree of erosion; the formed caverns merge, and the appearance of new ones is slightly slowed down as a result of the mechanical hardening of the impact layer by microjets and/or shock waves [36] and the damping effect of the water that penetrated the caverns during the compression phase of the sonotrode of the mechanical vibrating system [3,55].

The images in Figure 4 and Figure 5 are used for the fractographic analysis.

The SEM images (Figure 4) show a mix of caverns from very small to significant sizes, created by brittle and ductile fracture. In both cases, the effect of aluminum alloying is noticeable, through the deformations prior to cracking and fracture. In both situations, the relief of the cavitation-eroded surface is irregular in which large caverns are concentrated in the central area of the surface. Measurements made using the scanning electron microscope and the computer tomography show that the largest length and depth dimensions are at the 250/12 h structural state—up to 400 μm for length and up to 656 μm for depth—compared to the 250/24 h structural state—up to 300 μm for length and up to 645 μm for depth. The space between the red and yellow lines, light in color (towards white) represents the hardened layer, whose dimensions are 98 μm (state 250/12 h) and 91 μm (250/24 h state). These differences, associated with the microstructural refinement, intermetallic particle distribution, and microstructuralstability, confirm the higher resistance to cavitation erosion of the surface structure of the state 250/24 h, as also resulted from the analysis performed based on the photographic images in Figure 3. This confirms the higher resistance of the 250/24 h state. The fractographic investigation showed that, according to the shape of the caverns, the crack initiators are in the intermetallic compounds Al_8_Mn_5_, mainly, and the secondary phase β(Mg_17_Al_12_). According to the shape of the cracks generated in the depth of the surface structure and the ruptures (see images in Figure 5), it follows that they were preceded by plastic deformations due to the high tenacity of magnesium and its ability to absorb water. It is observed that, regardless of the structural state, the tunnel/cave-like shapes of some caverns, made perpendicular and inclined to the cavitated surface. We consider these shapes to be generated by an explosion-like effect of the water entering the caverns, at the overpressure exerted by the cavitation microjet which expels both groups of grains of the solid solution α, and pieces of grains of intermetallic compounds and of the secondary phase β. In conclusion, we consider that the similarities between the erosion morphologies of the two states are attributed to microstructural refinement, intermetallic particle distribution, and microstructural stability, and the differences are an effect of the microhardness values resulting from the artificial aging heat treatment regime together with the work hardening of the exposed surface layer.

### 3.2. Diagrams and Specific Parameters for Cavitation Behavior and Resistance

**Figure 6:**
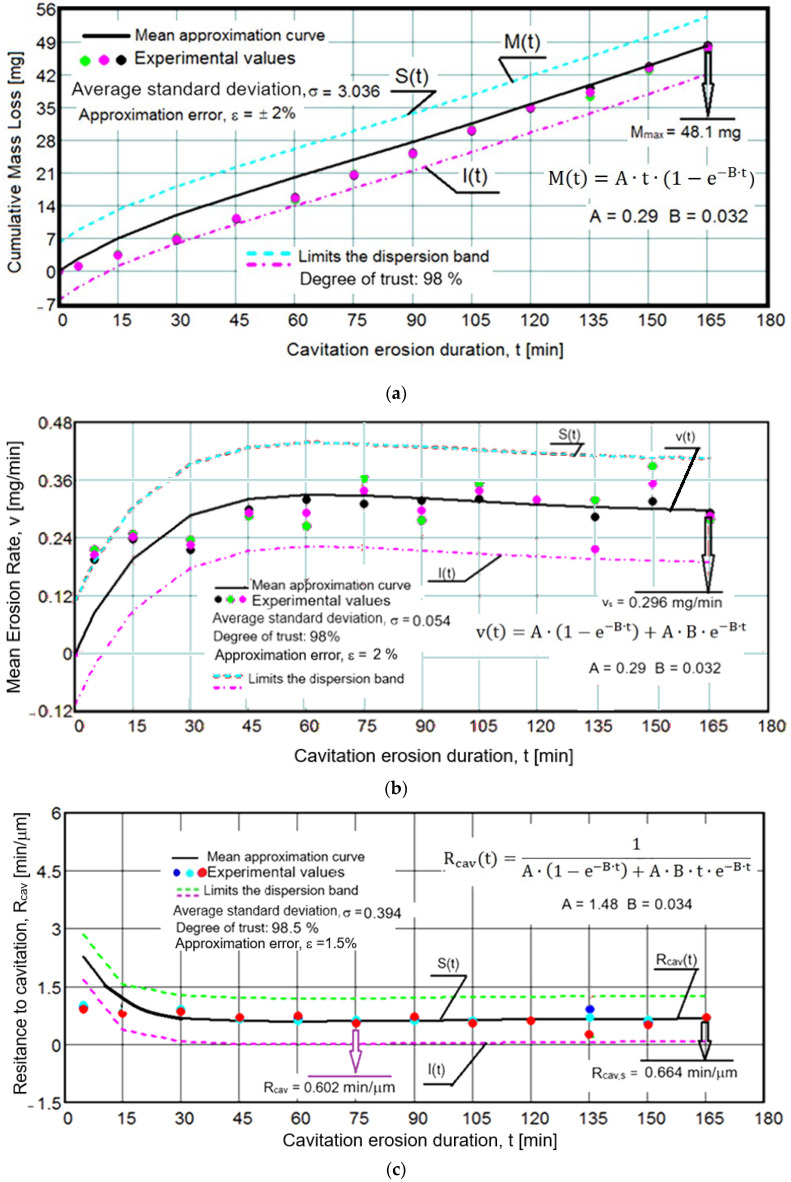
State-specific diagrams 250/12 h: (**a**) the variation of cumulative mass losses with cavitation duration (250/12 h); (**b**) the variation of erosion speed with cavitation duration (250/12 h); (**c**) the influence of cavitation attack duration on the strength of the stressed surface (250/12 h).

**Figure 7:**
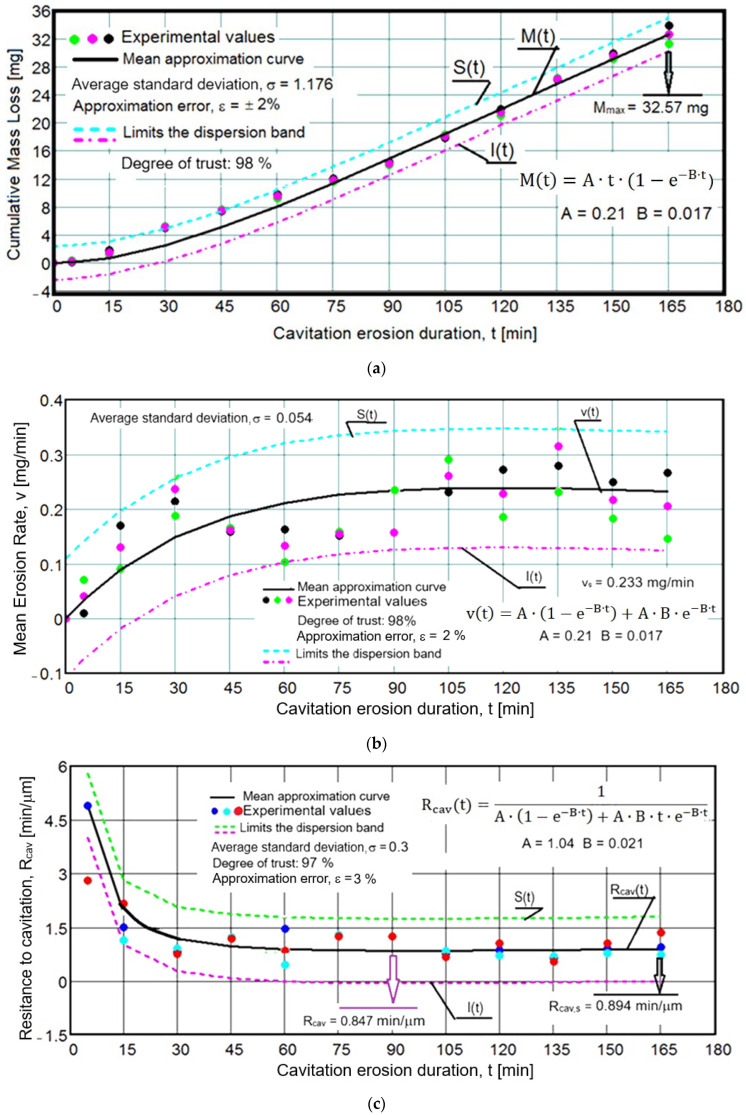
State-specific diagrams 250/24 h: (**a**) the variation of cumulative mass losses with cavitation duration (250/24 h); (**b**) the variation of erosion speed with cavitation duration (250/24 h); (**c**) the influence of cavitation attack duration on the strength of the stressed surface (250/24 h).

The specific diagrams and parameters used in discussions and evaluations of cavitation behavior are in accordance with the indications of ASTM G32-2016 [56] and laboratory custom [55]. These diagrams are presented in Figure 6 and Figure 7 and contain: the experimental values (marked by green, pink and black dots), their averaging curves with the statistical relationships used [55,57,58], the values of the parameters that allow the evaluation of the cavitation resistance of the surface structure and the values of the statistical parameters that provide confidence in the accuracy of the experiment [55]. The dispersion band of the experimental values, compared to the averaging curve, is delimited by the curves S(t) and I(t) that define the degree of confidence and, respectively, the approximation error ε. These curves are constructed using statistical relationships based on the standard deviation σ and have the forms [50]:

S(t) = [M(t), v(t), R_cav_(t)] + ε∙σ;   I(t) = [M(t), v(t), R_cav_(t)] − ε∙σ (1)

The form of the calculation relationship for the average standard deviation depends on the parameter being referred to:

(2) $$\sigma ={\left[\frac{\sum_{i=1}^{n}{\left(M_{i}-M(t{)}_{i}\right)}^{2}}{n-1}\right]}^{\frac{1}{2}},\sigma ={\left[\frac{\sum_{i=1}^{n}{\left(v_{i}-v(t{)}_{i}\right)}^{2}}{n-1}\right]}^{\frac{1}{2}},\sigma ={\left[\frac{\sum_{i=1}^{n}{\left(R_{cavi}-R_{cavi}(t{)}_{i}\right)}^{2}}{n-1}\right]}^{\frac{1}{2}}$$

Here, Mi is the cumulative mass lost through erosion; v_i_ is the erosion speed; R_cavi_ is the resistance to cavitation erosion defined as the inverse of the mean depth erosion rate; and n is the total number of intermediate periods (n = 12). All these are experimental values, calculated based on the mass losses (Δmi) recorded during the intermediate period “i”, as follows:

(3) $$M_{i}=\Sigma_{i=1}^{n}\Delta m_{i},\;v_{i}=\frac{\Sigma_{i=1}^{n}\Delta m_{i}}{\Sigma_{i=1}^{n}\Delta t_{i}},\;R_{cav_{i}}=\frac{\rho \cdot \pi \cdot d_{p}^{2}\cdot {\Delta t}_{i}}{4\cdot {\Delta m}_{i}}$$

M(t)_i_, v(t)_i_ and R_cavi_(t) are values defined by the averaging curves at time “i”; ρ = 1730 Kg/m^3^ is the alloy density.

As can be seen in the legends of these diagrams, the parameter values σ = 0.054 … 3.036 aresuperunitary values for approximating cumulative mass losses and sub-unitary values for approximating erosion velocities and cavitation resistances. These values determined very high confidence levels of 97% … 98.5%, corresponding to cumulative mass loss approximation errors of ±2%, certifying the accuracy of the experiment.

The analysis of the data in the diagrams presented in Figure 6 and Figure 7 shows, through similarities and differences, the effects of the microstructure (through the dimensions, number and dispersion of brittle intermetallic compounds and the shape and dimensions of the grains of the solid solution α and the solution of the secondary phase β) and of the hardness of the cavity surface.

The similarities consist in:

- The mode of scattering and dispersion of the experimental values with respect to the averaging curves M(t), v(t) and R_cav_(t). Correlated with the results published in [2,3,4,5,39,50,59], we appreciate that this mode is the effect of the alloy structure (solid solution with insertions of brittle intermetallic compounds).
- The shape of the averaging curves, also supported by the photographic images in Figure 4 after 75(90) min, by the research carried out in our laboratory [2,3,49,50] and by those in the literature [27,28,29,30,54,60], are effects of the microstructure and of the mechanical increase in hardness compared to the initial value, by the impact with shock waves and microjets.
  (a) Approximately linear increase in cumulative mass losses (M(t)), after the accumulation period 0 … 45(60) min.
  (b) Asymptotic decrease in the erosion velocities (v(t)) towards the value v_s_;
  (c) Exponential decrease followed by a slight linear increase in the cavitation resistances (R_cav_(t)).

The differences are between:

- The values of the parameters characterizing the surface (structural state 250/24 h), with the highest resistance to cyclic stresses of shock waves and cavitational microjets: M_max_ = 32.57 mg, v_s_ = 0.233 mg/min, R_cav,s_ = 0.894 min/μm. These values reflect the positive effect of the difference in initial hardness (about 5%) on the surface resistance to cavitation erosion (64 HV_5_ for 250/24 h versus 61 HV_5_ for the 250/12 h state).
- The slopes of the M(t) curves and the durations of the cavitation attack from which the averaging curves v(t) show a maximum, and from which the cavitation resistance R_cav_ starts to increase: t = 90 min for 250/24 h and t = 75 min for the 250/12 h state. These differences, from our and older studies [27,28,29,30,54,60] show the capacity of structures to absorb energy through plastic deformation and increase in hardness, mechanically through impacts, in addition to the initial one.

The erosion speed and cavitation resistance diagrams (Figure 6b,c and Figure 7b,c) show the relationship between erosion speed and the surface resistances to cavitation erosion. The times at which the v(t) curves start to decrease asymptotically towards the v_s_ values are identical to those at which the R_cav_(t) curves start to increase towards the R_cav,s_ values (75 min for the 250/12 h state and 90 min for the 250/24 h state). This relationship reconfirms the results of experimental studies [2,3,4,30,39,46] regarding the mechanical response of energy-absorbing structures through hardening under the cyclic stresses of vibrating cavitation, after a period of stress. This mechanical hardening is quantifiable through the increases in cavitation resistance ΔR_cav_ = ((R_cav,s_ − R_cav,i_)∙100/R_cav,i_), as shown in Table 1, and is reflected by the evolution of R_cav_(t) curves.

A pronounced increase can be observed at the 250/12 h state and a small one at the 250/24 h state. So, the greatest hardening is achieved in softer surfaces, with lower initial hardness. The findings resulting from the analyses of the specific diagrams in Figure 6 and Figure 7 are supported by the macro images in Figure 3 through the evolution of the caverns after 75 min and 90 min, respectively, in which the v(t) curves decrease approximately linearly towards the v_s_ value, while the R_cav_(t) curves increase slightly towards the R_cav,s_ value.

## 4. Cavitation Resistance Evaluation

**Figure 8:**
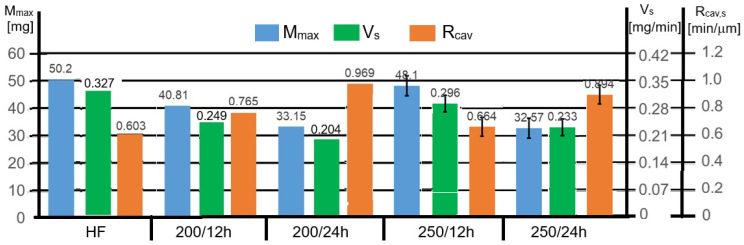
The histogram of comparison of parameters specific to the structure’s resistance to cavitation erosion.

To evaluate the differences in resistance to erosion generated by vibratory cavitation, the histogram in Figure 8 was constructed, using the values of the parameters recommended by ASTM G32-2016 standards and used in the practice of our research, displayed in the legends of Figure 6 and Figure 7 as well as those presented in [49], obtained for the semi-finished states (SF notation) and the structural states obtained by artificial aging at 200 °C with holding times of 12 and 24 h (symbolization 200/12 h, 200/24 h, respectively) [61].

The comparative analysis of the histogram data shows:

- 250/24 compared to SF: M_max_ decreases by about 54%, v_s_ speed decreases by about 40%, cavitation resistance R_cav,s_ increases by about 48%;
- 250/12 compared to SF: M_max_ decreases by about 4%, v_s_ speed decreases by about 10%, cavitation resistance R_cav,s_ increases by about 10%;
- 250/24 compared to 250/12: M_max_ increases by about 18%, v_s_ speed increases by about 19%, cavitation resistance R_cav,s_ increases by about 15%;
- 250/24 compared to 200/24: M_max_ decreases by about 2%, v_s_ speed decreases by about 14%, cavitation resistance R_cav,s_ increases by about 8%;
- 250/12 compared to 200/12: M_max_ increases by about 2.1%, v_s_ speed increases by about 19%, cavitation resistance R_cav,s_ decreases by about 15%;

These evaluations show that, compared to the semi-finished state (SF), both treatments lead to structures with superior strength. Compared to the 200/24 h structural state, the resistances to erosive cavitation stresses are lower. Compared to the 200/12 h condition, only the 250/24 h condition gives the surface greater resistance.

In relation to temperature and duration, the histogram shows that the temperature of 200 °C is better than that of 250 °C, and as a duration of maintenance at the heat treatment temperature, the 24 h condition offers the best resistance.

## 5. Conclusions

The artificial aging heat treatments at 250 °C with holding times of 12 h and 24 h, respectively, compared to the SF of elaboration (semi-finished product), according to the values of the reference parameters (R_cav_, M_max_, v_s_) recommended by the ASTM G32-2016 standards, represent a solution for increasing the cavitation resistance of the AM50 alloy structure to cavitation. This is determined by the increase in the surface microhardness value and the reduction in the grain sizes of the solid phases (α and β) and the dispersion and sizes of the hardening phases (AlMn).

The microstructures resulting from the two artificial aging heat treatment regimes (250/12 h and 250/24 h) do not show any significant changes between the grain sizes of the solid solutions (α and β); microscopic analyses showing differences between the dispersion and maximum dimensions of the brittle intermetallic phases (AlMn). For this reason, the cavitation behavior of the surfaces of the two structural states shows some similarities; the differences being given by the resistance to overpressures resulting from the impact with shock waves and cavitational microjets, also influenced by the initial hardness of the surface.

The surface degradation during the attack, through the appearance of caverns, their shape and dimensions (see Figure 3), expresses the dependence of the surface resistance to cavitation stresses and the alloy’s ability to absorb the pressure energy developed by the impact with the cavitational microjets/shock waves. The tunnel/cave shapes of some of the caverns (Figure 4 and Figure 5) suggest an explosion mechanism generated by the compression of the air/water in the caverns at the moment of impact with the cavitational microjets.

In terms of cavitation resistance, the artificial aging heat treatment at 250 °C for 24 h provides a resistance higher by about 15% compared to the 250/12 h state. Compared to the structural condition resulting from the 200 °C temperature regime for 24 h, the cavitation resistances of the 250/24 h and 250/12 h states are lower by (15 … 45)%.

The AM50 alloy heat treated by artificial aging at 250 °C, with durations of 12 and 24 h, is recommended in the manufacture of parts that work in conditions with reduced intensity of cavitation erosion. Such parts can be stents and valves used in cardiac repair surgery, where the blood circulatory system, from the point of view of flow, also has specific cavitation phases.

The study opens new directions of research into the behavior and resistance to cavitation of AM50 alloy with other structural states, resulting from the use of other heat treatment regimes or by hardening with modern remelting methods.

## Figures and Tables

**Table 1:** Increasing surface resistance through mechanical stress of cavitation.

Structural State	250/12 h	250/24 h
Resistance increase, ΔR_cav_, %	10.3	5.54

## Data Availability

The original contributions presented in this study are included in the article. Further inquiries can be directed to the corresponding authors.
